# Rare Unicentric Intrapulmonary Castleman Disease: A Systematic Review and Report of a Case

**DOI:** 10.2174/0118743064348696250107092627

**Published:** 2025-02-18

**Authors:** Manjinder Kaur Pannu, Jonas Peter Ehrsam, Olga Meier Adamenko, Ilhan Inci, Othmar Markus Schöb

**Affiliations:** 1 Medical School, University of Nicosia, Nicosia, Cyprus; 2 School of Medicine, University of Zurich, Zurich, Switzerland; 3 Thoracic Surgery Clinic, Klinik Hirslanden Zürich, Zürich, Switzerland

**Keywords:** Castleman disease, Thoracic surgery, Intrapulmonary castleman disease, Unicentric castleman disease, Lymph node tissue, Disorder

## Abstract

**Objectives:**

Castleman disease (CD) is a very rare B-cell lymphoproliferative disorder marked by the abnormal enlargement of lymph node tissue. It can present as either unicentric (UCD) or multicentric, with the former often appearing in intrathoracic regions, although its presence within the lungs is uncommon.

**Methods:**

We report the case of a 42-year-old woman who underwent resection of an 11 cm intrapulmonary UCD. Additionally, we conducted a systematic review of the demographics, clinical presentation, diagnosis, and treatment approaches for intrapulmonary UCD.

**Results:**

Our review identified 35 documented cases of intrapulmonary UCD, including our case. The average age was 34 years, with a female predominance of 57.7%. Tumor sizes ranged from 1.5 to 11 cm, with our case being the largest. Of the 24 cases with reported anamnesis, 58.3% were asymptomatic, while 41.7% had nonspecific symptoms such as cough, chest pain, or fever (as in our case). Histological analysis was available for 24 cases, with 83.3% identified as the hyaline vascular type. Biopsies through small needle aspiration or fresh-frozen samples failed in all attempts, requiring resection for diagnosis and treatment. Due to high vascularity, delicate location, and lack of diagnosis, lobectomy or pneumonectomy was performed in 45.7% of cases. Among the 11 cases with reported follow-up, no disease recurrence was observed over an average of 3 years.

**Conclusion:**

Our systematic review highlights the rarity of UCD in the lungs. The demographics of intrapulmonary UCD align with the general disease profile. Surgical removal is crucial for both diagnosis and treatment. The significant vascularity and pulmonary location of these tumors present challenges, requiring pre-operative awareness and precautions.

## 1 INTRODUCTION

Castleman disease (CD) is a B-cell lymphoproliferative disorder marked by the abnormal growth of lymph nodes and, less commonly, extra-nodal locations (5%) [1]. Its rarity is underscored by an estimated 21 to 25 cases per million person-years, classifying it as an orphan disease [2].

CD presents in two distinct subtypes: the more common unicentric CD (UCD), usually asymptomatic, and the less common multicentric CD (MCD), which is often severe and systemic [3, 4]. Histologically, UCD can be divided into three main forms: a) hyaline vascular (HV) variant (76-91%), b) plasma cell (PC) variant (10-24%), and c) mixed variant (1-4%) [5].

The exact pathogenesis of CD remains unclear, but it is generally thought to result from impaired immunoregulation. Some theories suggest that UCD originates from an antigen stimulus affecting an abnormal plasmacytoid monocyte population in a lymph node, whereas MCD is believed to be triggered by factors such as chronic infection, chronic low-grade inflammation, viral infections, abnormal cytokine modulation, and, in some cases, infections with human herpesvirus-8 and HIV [3, 5].

The frequency of UCD is reported to be about 31% in the thorax (31, 29, 89%, respectively), 23% in the cervical region (6, 23, 42%, respectively), 18% in the abdomen (18, 38, 1%, respectively), with the remainder occurring more peripherally [1, 6, 7]. In intrathoracic cases, most affect either the mediastinum or the hilum [1, 6-10]. However, intrapulmonary locations, including those in the interlobar fissure, parahilar, and fully intraparenchymal areas, are considered exceptionally rare.

In this report, we provide a comparative analysis by systematically reviewing the limited previously published literature on intrapulmonary UCD. Additionally, we also present a rare case of intrapulmonary UCD.

## 2 CASE PRESENTATION

A 42-year-old Caucasian woman with a history of hay fever, asthma, metabolic syndrome, and prior radiotherapy for thyroid adenoma consulted her general practitioner due to a persistent cough and fever lasting three weeks. During the consultation, her thyroid and inflammatory markers appeared normal, but lung auscultation revealed rales in her left hemithorax. A chest x-ray showed an indistinct contour in the middle left lung area.

**Fig. (1):**
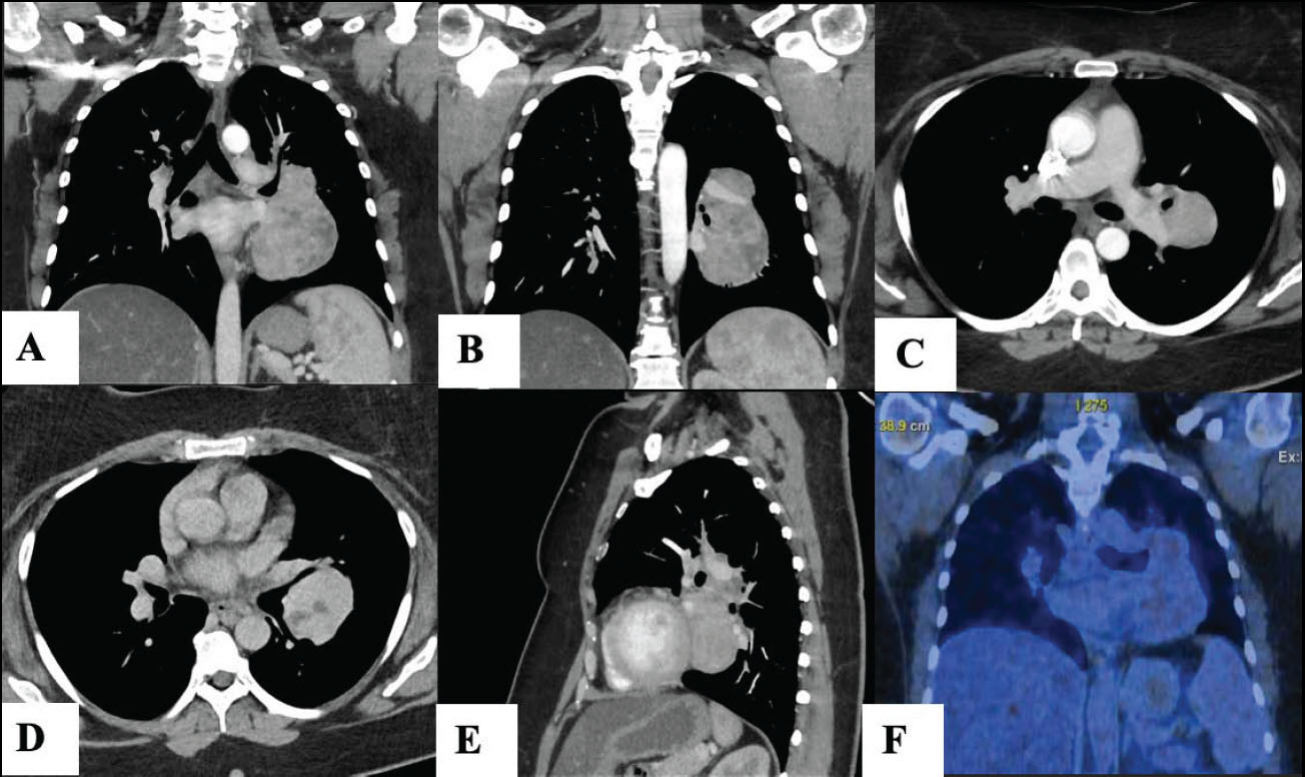
CT scan of the Unicentric Castleman tumor **A**) hemi-encircling the left upper lobe bronchus in coronal view, **B**) the inflow into the upper- and lower pulmonary vein in coronal view, **C**) the attachment and nutritive branch from the pars interlobaris of the pulmonary artery in axial view, **D**) the broad attachment in the bifurcation of the upper and lower lobe bronchus in axial view, **E**) the location with neighboring vessels and bronchi in sagittal view. **F**) PET-CT scan showing slight metabolic activity in the tumor.

A subsequent thorax CT scan (Fig. **1A**-**D**, **F**) revealed an 11 x 8 x 5 cm partially necrotic mass with cystic, adipose, slightly calcified characteristics, and contrast agent uptake. The mass extended from the mediastinum along the left hilus, the junction of the left main bronchus, and into the left lung. There was an extensive connection between the pars interlobaris of the pulmonary artery and the upper and lower pulmonary veins on the left side. Although there was no apparent direct invasion of these blood vessels, potential diagnoses included primary lung cancer, angiosarcoma, ectopic thyroid tissue, or a teratoma (though the location was atypical).

A PET-CT scan with FDG enrichment showed minimal metabolic activity within the mass (Fig. **1E**), making lung cancer, angiosarcoma, and thyroid tissue involvement less likely. This shifted the differential diagnoses toward a neuroendocrine tumor or a solitary fibrous tumor of the pleura. A transbronchial needle biopsy was non-conclusive, revealing fibrotic, non-malignant cells.

**Fig. (2):**
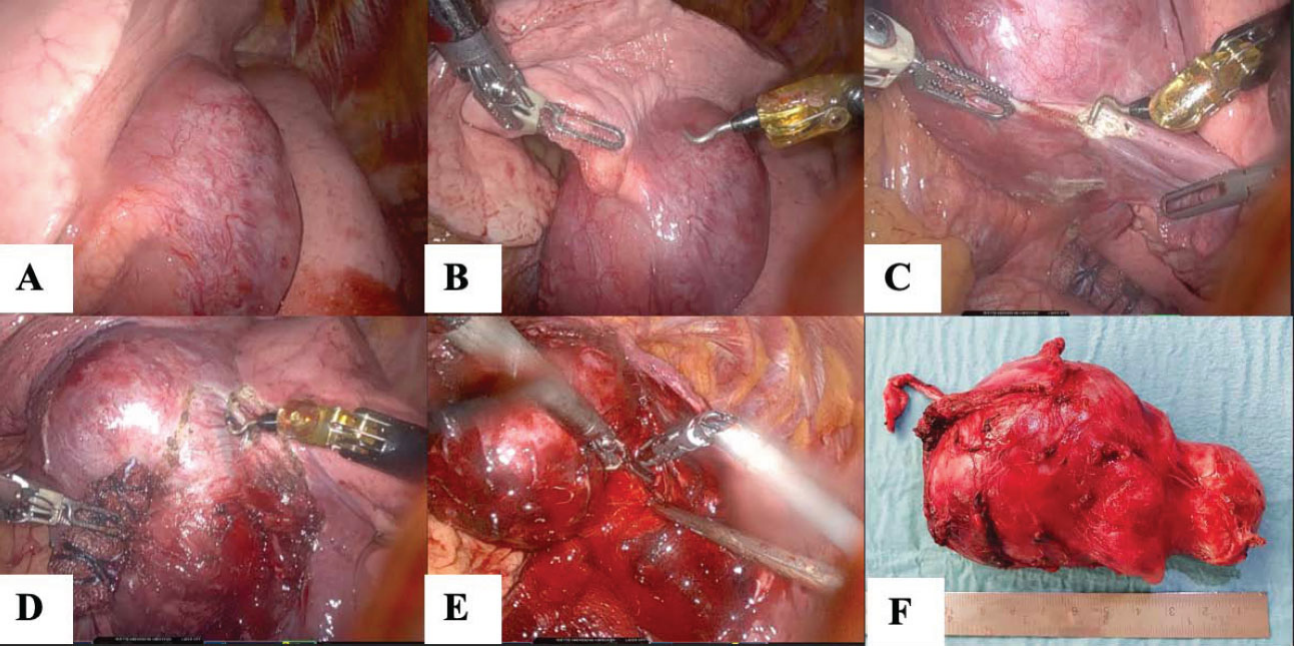
Unicentric castleman tumor during robotic assisted thoracic surgery **A**) view on interlobar fissure, **B**) resection from upper lobe, **C**) resection from lower lobe, **D**) resecting branches draining into the lower lobe veins, **E**) view on the pars interlobaris of the pulmonary artery in the opened interlobar fissure. **F**) Resected histological specimen.

Following an interdisciplinary board discussion, the patient underwent a diagnostic DaVinci robot-assisted thoracoscopy (Fig. **2A**-**E**). During the procedure, it was discovered that the tumor was unattached to the mediastinum and hilum but located in the pulmonary fissure, attached by lung tissue from the upper and lower lobes, partially encircling the upper lobe bronchus. It was supplied by a branch from the pulmonary artery and bronchial artery and drained into the upper and lower lobe veins. A frozen section biopsy failed to provide a conclusive diagnosis, as it contained some lymphatic cells, which could potentially indicate a lymphatic neoplasia. Consequently, the surgical team proceeded with the resection and mobilization of the mass while preserving its lung tissue capsule.

Due to significant vascularity toward the center of the fissure and insufficient exposure for precise vessel ligation, the surgical approach was converted to a thoracotomy. The tumor was completely removed while sparing all lung tissue. However, due to ongoing bleeding, with a total blood loss of 2000 ml, a large hemostasis patch (Veriset^TM^ 8x16cm) was applied to the wound bed, resulting in rapid and successful hemostasis. The entire surgical procedure concluded after 145 minutes.

Following the surgery, the patient received a transfusion of 2 units of red blood cells. She was extubated just one hour after the procedure and transferred from the intensive care unit to the regular ward the following day. Her subsequent hospital stay was without significant complications, and she was discharged on the sixth day post-operation.

**Fig. (3):**
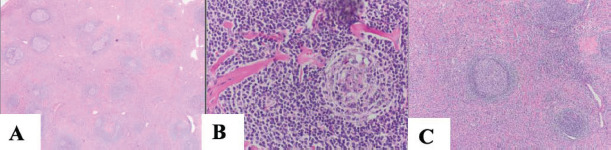
Histological slides of the resected unicentric Castleman tumor of the hyaline vascular type: **A**) overview (HE x40), **B**) a typical hyalinized vessel radially penetrating the germinal center (lollipop sign) (HE x100), **C**) lymphoid follicles with atrophic germinal centers showing proliferation of follicular dendritic cells (HE x100).

The removed surgical specimen weighed 133 grams (Fig. **2F**). The conclusive histological examination (Fig. **3A**-**C**) revealed a large lymph node with lymphoid tissue showing angio-follicular hyaline hyperplasia, numerous regressed germinal centers, interstitial vascular proliferation, and increased interstitial fibrosis. Immunohistochemical studies showed no signs of lymphoma. After consultation with a second pathologist, the diagnosis of CD, specifically the localized hyalin vascular variant, was confirmed. There was no evidence of malignancy, and the resection was considered radical and in healthy tissue.

Since there was no radiological evidence of additional lesions, it was determined that this form of CD was unicentric. The complete removal of this single lesion was deemed curative, so the interdisciplinary medical board decided against further treatments, opting for regular follow-ups instead.

During the first follow-up after six months, a CT scan showed no signs of relapse, and the patient remained symptom-free. It was then decided to extend the interval, until the next follow-up, to one year.

## 3 METHODS

In April 2024, a systematic review was conducted in accordance with the PRISMA guidelines. The PubMed database was used to identify case reports and case series that evaluate the demographics, clinical presentation, diagnosis, and treatment strategies of intra-pulmonary UCD. Additionally, a non-systematic search of the Google Scholar database was conducted using the same keywords.

The following search string was used:

((“Castleman”[tiab] OR “Morbus Castleman”[tiab] OR “Castleman’s disease”[tiab] OR “Castleman disease “[tiab] OR “lymphoid hamartoma”[tiab] OR “lymph node hyperplasia”[tiab]) AND (“lung”[tiab] OR ” pulmonal”[tiab] OR “intrapulmonal”[tiab] OR “intra-pulmonal”[tiab] OR “pulmonary”[tiab] OR “intra-pulmonary”[tiab] OR “intra-pulmonary”[tiab] OR “bronchial”[tiab]))

Promising original articles and reviews were also scrutinized for any additional relevant articles listed in their references. The inclusion criteria focused on intrapulmonary CD without restrictions regarding patient age, publication year, or language; all foreign language articles were translated and assessed *via* Deepl.com. Cases of MCD with pulmonary lesions and cases of UCD reported at the pulmonary hilus or mediastinum were excluded.

The database search and individual article reviews were independently conducted by both M.K.P. and J.E.P. to prevent selection bias. The studies identified were independently organized according to the specific sub-criteria outlined in Table **1**. These tabulated studies were then compared to identify any potential disagreements.

The results are presented as means and percentages, either for the total number of identified cases or for specific subgroups or sub-criteria. Studies that lacked data on a particular sub-criterion were excluded from the analysis of that sub-criterion.

## 4 RESULTS

The systematic review conducted in PubMed yielded 318 publications from 1970 to 2024. After screening titles and abstracts, 81 articles were selected for further examination, including their reference lists. Forty-four studies with a total of 132 reported cases of MCD with pulmonary lesions were excluded, along with 11 records that did not meet the pulmonary UCD criteria. Two additional records were identified through reference lists. No conflicts or disagreements arose between the two authors who independently reviewed the database.

**Fig. (4):**
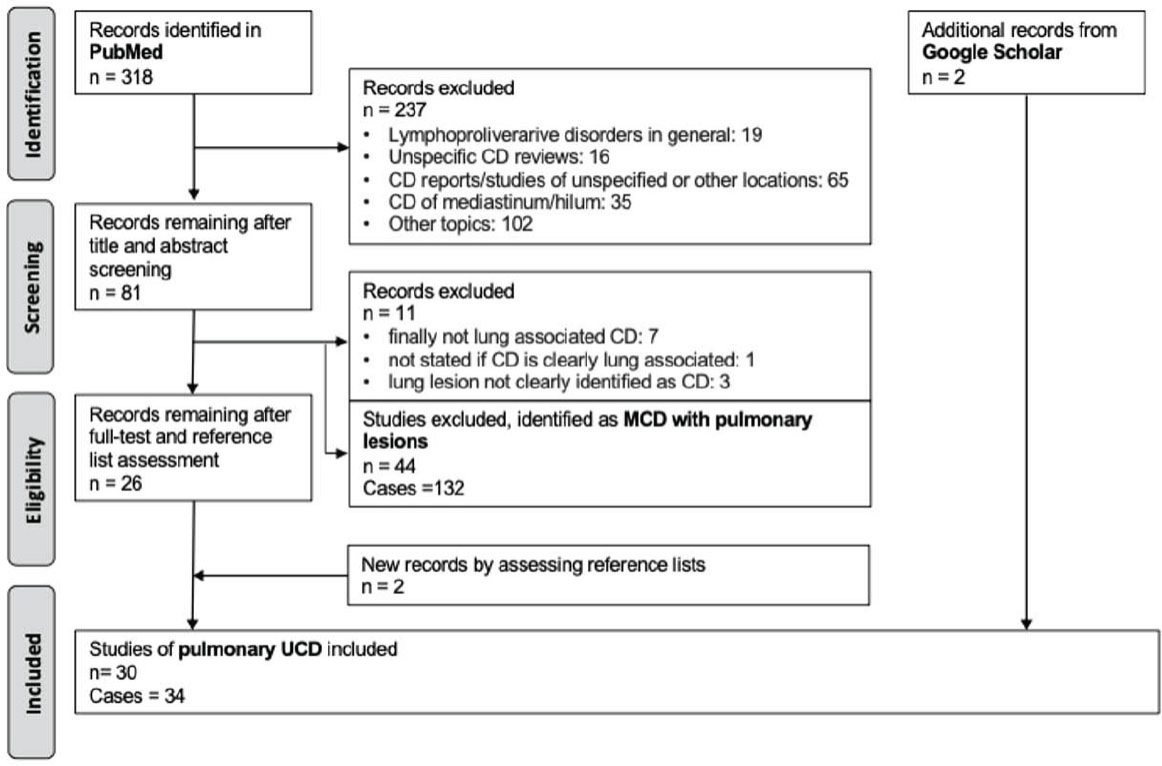
PRISMA diagram investigating resources on unicentric Castleman disease. A total of 318 articles were retrieved from PubMed. During the initial screening process, 237 articles which did not meet the inclusion criteria were excluded, leaving 81 articles included. A second screening process resulted in 11 and 44 articles being excluded, leaving 26 full length articles. During the final phase of evaluation, a total of four extra articles were discovered from reference lists and Google Scholar, resulting in 30 pulmonary UCD articles and 34 cases.

Additionally, a non-systematic search of the Google Scholar database revealed two more records. This process resulted in a total of 30 articles reporting 34 cases. The literature selection process is displayed in the PRISMA flow diagram (Fig. **4**). Table **1** summarizes the collected cases.

The reporting among the 34 cases was highly heterogeneous. Complete datasets that met all the desired sub-criteria listed in Table **1** were available for only 7 of the 34 cases. The analyses of these sub-criteria are presented in the discussion section.

## 5 DISCUSSION

Intrapulmonary UCD is rare. Our presented case, along with the systematic review, revealed a total of only 35 documented intrapulmonary UCD cases (Table **1**).

### 5.1 Prevalence/Incidence

Estimating the frequency of intrapulmonary UCD within the broader context of all UCD localities is challenging due to its status as an orphan disease. However, intrapulmonary UCD appears to occur in a low single-digit percentage range. For instance, Keller *et al*. (1972) [6] reported an intrathoracic occurrence of 89.2% (66/74) in a large series of 74 UCD patients, but only 2.7% (2/74) had isolated lung involvement. In contrast, a systematic review by Talat *et al*. (2012) [7] found a 28.1% intrathoracic occurrence in 235 cases, with no instances of lung involvement. Similarly, Danon *et al*. (1993) [1] reported a 31% intrathoracic occurrence among 102 patients but no lung involvement. Additionally, several other studies reflect the rarity of intrapulmonary UCD. McAdam *et al*. (1998) [8] reported a 4.2% (1/24) intrapulmonary occurrence in their series on intrathoracic UCD. Lou *et al*. (2015) [9] found a 12.5% (2/16) occurrence, and Xioxian *et al*. (2020) [10] reported an 11.1% (2/22) occurrence in their respective case series. These findings collectively indicate that intrapulmonary UCD is indeed quite uncommon.

### 5.2 Histology

Among the 35 identified intrapulmonary UCD cases, 24 had clearly reported histological subtypes and seven more were likely of the HV subtype. Of the cases with clear histology, 83.3% (20/24), were of the HV type, including our case, 12.5% (3/24) were of the PC type, and 4.2% (1/24) were of the MC type. The predominance of the HV type was also observed in the case series by McAdam *et al*. (1998) [8] and Lou *et al*. (2015) [9], which reported 95.8% and 75% HV type for various intrathoracic localities, respectively. This aligns with the typical histology of UCD in general, estimated to be 77.7% in the systematic review by Talat *et al*. (2012) [7] and even 91% in the large case series by Keller *et al*. (1972) [6].

### 5.3 Symptoms

In general, UCD is often reported to be asymptomatic (51-66.6%) [4, 6] or to present with flu-like symptoms [5, 11]. Among the 35 compiled cases of intrapulmonary UCD (Table **1**), only 24 cases provided information on symptom anamnesis. Of these, 14 (58.3%) were entirely asymptomatic, while 10 (41.7%) exhibited nonspecific symptoms such as cough (N=7/10), fever (N=2/10), dyspnea (N=2/10), and chest or back pain (N=4/10). The coughing symptoms may be partly explained by compression and irritation of the adjacent bronchial system or post-stenotic infection, and the pain may be due to pressure on the pleura parietalis. In three cases, the intrapulmonary lesion was observed for 1 [12], 3 [13], and even 7 years [14] before resection, showing varying growth dynamics from very slow to tripling in size within a year. The HV subtype's ability to grow slowly over many years has been described in other localities [1].

### 5.4 Location

No side specificity for intrapulmonary UCD was detected in our literature review. Of the documented cases, 13 UCD lesions were located in the right lung and 14 in the left lung, while the rest did not specify. Regarding the location, 9 cases were described as fully embedded in the periphery of the lung parenchyma, 13 cases had lesions originating parahilar, proximal on a lobe bronchus, sometimes expanding into the interlobar fissure as in our case, 11 cases originated in the interlobar fissure itself, and 2 cases did not provide details.

### 5.5 Lesion Size

Among the cases listed in Table **1**, the average size of the pulmonary lesions was 4.62 cm, with sizes ranging from 1.5 cm to 11 cm. Our reported case is noteworthy as it stands out as the largest intra-pulmonary CD reported to date. Such a large size of UCD appears uncommon compared to the documented growth potential in other body locations. In large series, Danon *et al*. (1993) [1] and Keller *et al*. (1972) [6] reported medians of 7 cm and 5.8 cm and maximum sizes of 10 cm and 16 cm, respectively. Talat *et al*. (2012) [7] detected a mean size of 5.5 cm in their large review of intrathoracic cases, with a maximum of up to 20 cm, while McAdam *et al*. (1998) [8] even reported an outstanding intrathoracic lesion size of 25 cm.

### 5.6 Age

Among our compiled intrapulmonary UCD cases, the average age at presentation was 34.0 years, ranging from 16 to 72 years. Generally, UCD can occur at any age [5], but our finding aligns closely with the mean age of 33.8 ± 17.8 years found in a systematic review of 278 cases [7] from various locations, the mean age of 30.5 ± 8 years in a case series of 32 intrathoracic UCD cases [9], and the mean age of 34 years in a case series of 24 intrathoracic UCD cases [8].

### 5.7 Gender

Regarding gender in our detected intrapulmonary UCD cases, 15 were identified as female and 11 as male, while gender was not stated in 9 cases. Excluding the unstated group, the proportion was 57.7% female to 42.3% male. Female dominance was also reported in various UCD locations in the review by Talat *et al*. (2011) [7], which found 59.4% (165/278) female, and the case series of Danon *et al*. (1993) [1], which reported 66% (71/93) female. Similarly, the intrathoracic series by McAdams *et al*. (1998) [8] reported 75% (18/24) female [8]. However, the case series by Keller *et al*. (1972) [6] for various locations and the case series by Luo *et al*. 2015 [9] on various intrathoracic locations found no particular gender dominance, with the latter reporting an equal distribution of 50% (8/16) female.

### 5.8 Ethnicity

No race predominance was observed in our intrapulmonary UCD series. Of the collected cases, 42.8% (15/35) were of Asian origin, which aligns with the global population distribution. Similarly, Keller *et al*. (1972) [6] did not observe any obvious race predominance in their series involving various localities

### 5.9 Imaging

Among all intrapulmonary UCD reports, no typical pathognomonic characteristics were detected in CT, PET-CT, or MRI scans. This is a known issue for CD in general, leading to various differential diagnoses [7, 8, 15-17]. Additionally, only subtle correlations exist between radiological findings and histological types [15, 16, 18].

### 5.10 Diagnosis

Histopathological assessment of a large tumor biopsy is the gold standard for CD diagnosis [19, 20]. All six reported cases where an intrapulmonary UCD lesion was attempted to be diagnosed solely by bronchoscopic small needle aspiration or transthoracic biopsy were inconclusive. Notably, in five cases, including ours, intraoperative fresh-frozen sample diagnosis was also inconclusive. Furthermore, biopsies may pose a high bleeding risk due to the typically highly vascularized nature of CD tumors [21, 22], a scenario specifically reported in one case [18].

### 5.11 Management

Complete surgical resection is generally considered the gold standard for managing UCD [19, 23]. However, our review revealed that resecting intrapulmonary lesions is particularly delicate and demanding.

Out of the 35 compiled intrapulmonary UCD cases listed in Table **1**, truly lung-sparing, limited resections that preserved most of the lung parenchyma were clearly reported in only two cases (5.7%): the case of Sarana *et al*. (2017) [24] and our case.

At least 45.7% (16/35) of the intrapulmonary UCD cases required significant lung parenchyma resection. Specifically, 11 cases (31.4%) involved lobectomy, 3 cases involved either lobectomy or pneumonectomy and 2 cases definitely required pneumonectomy. However, there were likely more instances of significant resection that were not detected due to the lack of reporting.

The reasons for extensive lung resection were stated as follows: A) the necessity to be radical due to the inability to intraoperatively exclude pulmonary malignancy [14, 17, 25], B) the central intraparenchymal location of the lesion [26], and C) the rich blood supply of the HV type leading to severe intraoperative bleeding [18, 22, 24]. Excessive hemorrhage is a well-known issue reported from several lesions in other intrathoracic locations: Keller *et al*. (1972) [6] and McAdam *et al*. (1998) [8] reported 4 and 6 such cases, respectively. Both we and Sarana *et al*. (2017) [24] also encountered high vascularity, which necessitated abandoning the minimally invasive robotic and thoracoscopic methods, respectively, in favor of thoracotomy.

In only 6 of the compiled cases (17.1%) [10, 13, 22, 27, 28], the intrapulmonary UCD lesions, up to 5cm in size, were specifically described as having been removed * via* minimally invasive thoracoscopy. The other 82.9% of cases required thoracotomy for sufficient visibility. The challenge of successful minimally invasive resection for UCD lesions is not limited to the lung. Talat *et al*. (2012) [7] reviewed various intrathoracic UCD cases and found that only 3 out of 93 cases (3.2%) were operated on thoracoscopically. Similarly, Lou *et al*. (2015) [9] reported that only 1 out of 16 cases (6.3%) underwent thoracoscopic surgery.

Regarding the radicality of resection of intrapulmonary UCD, Sarana *et al*. (2017) [24] reported a subtotal resection due to bleeding and lung-sparing intentions. Most other intrapulmonary UCD reports did not specify their microscopic resection margins, but the number of subtotal resections might be high. For instance, McAdam *et al*. (1998) [8] reported subtotal resections in 10 out of 24 cases of intrathoracic, non-lung UCD localities due to the challenge posed by adjacent vessels or other delicate intrathoracic structures. As a consequence, surgeons should be mindful of the heightened risk of intraoperative severe bleeding, which is especially notorious in the hyaline vascular type. To mitigate such complications, preoperative angiography of the lesion to understand its blood supply is recommended [22], and angioembolization has occasionally been reported for intrathoracic, though not specifically for intrapulmonary lesions [29-31].

Rather than surgery, a few authors recommend radiotherapy as an alternative, particularly in cases where surgical intervention is impractical or if the lymph node is inaccessible [20, 23]. However, we did not find any instances of intrapulmonary UCD in our literature review that were treated exclusively with radiotherapy. Only one case [24], which was believed to have been removed non-radically, received adjuvant stereotactic radiation and showed no recurrence within at least six years. In a study involving 32 patients with UCD or MCD in various locations, treating solely with radiotherapy resulted in a complete response rate of only 43.8%, a partial response rate of 43.8%, and a non-response rate of 12.4%, with no observed dose-response relationship. The authors concluded that neoadjuvant radiotherapy might facilitate surgical resection of locally advanced or irresectable UCD disease. However, they did not recommend radiotherapy as an alternative to surgery [32].

### 5.12 Prognosis

The disease-free survival of intrapulmonary CD after resection was scarcely reported in our literature analysis. Only 11 out of 24 intrapulmonary UCD cases included follow-up data, ranging from 6 months to 14 years (mean 3.0 years), with no reported recurrences. A systematic review of 278 patients with UCD at various locations found that surgical tumor resection resulted in an impressive 95% disease-free survival rate at 3 years [7].

### 5.13 Intrapulmonary Multicentric CD

Of note, despite the rarity of UCD in the lung, our literature review frequently reported lung involvement in MCD. We identified 132 cases of MCD with pulmonary lesions, indicating that 21.0% of pulmonary Castleman cases are UCD, while 79.0% are MCD with intrapulmonary involvement. This suggests that pulmonary Castleman disease is more likely to be of the MCD type than the UCD type. A large case series on 162 patients with MCD found a pulmonary involvement rate as high as 35.8% [33]. However, considering the overall estimated occurrence of MCD at only about 23% [2], this is remarkable. It shows an opposite trend in the lung compared to other parts of the body, where UCD is more common (Table **1**).

### 5.14 Limitations

Our systematic review is limited by the small number of existing cases and further compounded by the heterogeneity and often incomplete reporting of sub-criteria. This limits us to making only approximate assumptions and introduces a remaining risk of bias in the evidence.

## CONCLUSION

Our systematic literature review highlights the extreme rarity of intrapulmonary UCD, with only 35 reported cases. In contrast, MCD with lung involvement is more frequent. Due to the limited number of cases and the often-incomplete reporting, comparisons and definitive conclusions are challenging. However, the demographic characteristics for intrapulmonary UCD appear to align broadly with the general disease profile. Resection is essential for diagnosis and is the gold standard for successful long-term therapy. The lung location is particularly challenging, and the high vascularity of these tumors must be considered to avoid significant blood loss and to achieve lung-sparing surgery.

## Figures and Tables

**Table 1:** Data sets representing the desired sub-criteria of all the cases.

**Study/Refs.**	**Location**	**Size (cm)**	**Histology**	**Diagnostics**	**Treatment**	**Age**	**Sex**	**Symptoms**	**Onset**	**Disease-Free Time**
**Current Case**	Parahilar LUL & interlobar fissure	11x8x5	HV	Transbronchial biopsy; intra-OP fresh-frozen section (non-diagnostic)	Lung resection (RATS switched to open)	42	F	Cough, fever	3w	≥ 6m
**Keller 1972 [6] A**	Parahilar, adjacent to a bronchus	NS	Likely HV	NS	Open lobectomy/pneumonectomy	NS	NS	NS	NS	NS
**Keller 1972 [6] B**	Parahilar, adjacent to a bronchus	NS	Likely HV	NS	Open lobectomy/pneumonectomy	NS	NS	NS	NS	NS
**Keller 1972 [6] C**	Intraparenchymal (coin lesion)	1.5	Likely HV	NS	Open lobectomy/pneumonectomy	NS	NS	NS	NS	NS
**Awotedu 1990 [34]**	Intraparenchymal LLL, segment 6	8x6	HV	Pleural biopsy (inflammatory changes)	Open pneumonectomy	21	M	Chest pain, fever, effusion	2w	≥ 3y
**Lee 1991 [35]**	Parahilar ML	NS	HV	NS	NS	NS	NS	NS	NS	NS
**Caus 1992 [36]**	Interlobar fissure	NS	NS	NS	NS	NS	NS	NS	NS	NS
**Spendini 1995 [37]**	Interlobar fissure ML & RLL	NS	NS	Transbronchial biopsy (non-diagnostic)	Surgical resection	51	F	NS	NS	≥ 3y
**Leung 1997 [28]**	Interlobar fissure ML & RLL	NS	HV	NS	VATS excision	30	F	Asymptomatic, anemia	-	≥ 19m
**McAdams 1998 [8]**	Parahilar LLL, interlobar fissure	~10	HV	NS	Open resection	28	M	Asymptomatic	-	NS
**Ferrozzi 2001 [15]**	Intraparenchymal LUL, segment 3	1.5	HV	NS	Tumor excision	46	F	Asymptomatic	-	NS
**Hosoda 2003 [12]**	Left interlobar fissure	NS	NS	NS	Open resection	21	M	Asymptomatic	-	NS
**Nishii 2004 [27]**	Right interlobar fissure	3x2x2	HV	NS	VATS removal	33	M	Asymptomatic	-	NS
**Minami 2005 [38]**	Left interlobar fissure	7x5.5x4.5	NS	NS	Open extirpation	19	F	Cough	NS	NS
**Mohanna 2006 [11]**	Intraparenchymal LUL, lingula	4x3.5	HV	No	Complete surgical resection	54	F	Back pain	1m	≥ 14y
**Yeh 2007 [25]**	Parahilar LUL	4.5x3	HV	No	LUL resection	42	M	Cough	2m	NS
**Kawczun 2007 [39]**	Interlobar fissure RUL & ML	3	HV	Pre-op angiography, intra-OP fresh-frozen section (malignancy suspected)	Open resection	40	F	Chest pain, dyspnea	20y	NS
**Yekeler 2009 [21]**	Interlobar fissure ML & RLL	3.5	HV	No	Open resection	43	F	Cough	6m	NS
**Racil 2009 [18]**	Interlobar fissure RUL & ML	4.4x2.8	HV	No	Pneumonectomy	23	F	Dyspnea, cough	4y	≥ 1y
**Tokunaga 2009 [14]**	Parahilar LLL	3.5	HV	No	LLL resection	23	F	Asymptomatic	known≥ 7y	NS
**Wang 2009 [40]**	Parahilar LLL, interlobar fissure	7.5	HV	No	Tumor excision	27	M	Asymptomatic	-	NS
**Günlüoglu 2011 [41]**	Parahilar RLL & interlobar fissure	5.5	PC	Transbronchial, transthoracal biopsy (inconclusive)	Open limited resection	29	M	Asymptomatic	-	NS
**Murinello 2011 [42]**	Intraparenchymal RLL, segment 6	2	PC	No	RLL resection	60	M	Asymptomatic	-	≥ 9m
**Ota 2013 [22]**	Parahilar RLL	5	HV	No	VATS resection RLL	19	M	Asymptomatic	-	≥ 8m
**Chiang 2013 [43]**	Intraparenchymal LUL, lingula	2.1x1.2	PC	NS	Wedge resection	72	F	Asymptomatic	-	NS
**Nadir 2014 [17]**	Parahilar RUL	6x5x4	HV	No	Open resection RUL	28	F	Asymptomatic	NS	NS
**Rawashdeh 2015 [13]**	Intraparenchymal LUL	4.8	HV	Intra-OP frozen section (able to rule out carcinoma)	VATS LUL resection	16	F	Chest pain, cough	3y	NS
**Liu *et al* 2014 [44]**	LUL (likely intraparenchymal)	3.1x2.9x3	HV	No	LUL resection	32	M	Asymptomatic	-	≥ 2y
**Luo 2015 [9]**	intrapulmonary fissure	NS	Likely HV	NS	Open resection	NS	NS	NS	NS	NS
**Luo 2015 [9]**	lung parenchyma	NS	Likely HV	NS	Open resection	NS	NS	NS	NS	NS
**Haager 2015 [26]**	Parahilar ML & interlobar fissure	3x2.4	HV	Transbronchial biopsy (non-diagnostic). Intra-OP frozen section (lymphoma/carcinoid appearing)	Open resection ML	24	M	Cough, bronchopulmonary infection	<1m	NS
**Cao 2015 [45]**	RLL	4.5x3.5x3	MC	Bronchoscopic biopsy (lymphocytic tissue)	Tumor excision	39	F	NS	2m	≥ 6m
**Sarana 2017 [24]**	Interlobar fissure RUL & ML	2.5x2.5x2	HV	No	VATS switched to lung-sparring thoracotomy	22	F	Asymptomatic	known≥ 2y	≥ 6y
**Xiaoxian 2020 [10]**	parahilar partially in lung parenchyma	NS	Likely HV	NS	VATS lobectomy	NS	NS	Asymptomatic	NS	NS
**Xiaoxian 2020 [10]**	Intraparenchymal	NS	Likely HV	NS	VATS lobectomy	NS	NS	Asymptomatic	NS	NS

**Abbreviations: **LUL: Left Upper Lobe. LLL: Left Lower Lobe. RUL: Right Upper Lobe. RLL: Right Lower Lobe. ML: Middle Lobe. HV: Hyaline Vascular. PC: Plasma Cell. MC: Mixed Cellularity. NS: Not Stated. VATS: Video-Assisted Thoracoscopy. RATS: Robot-Assisted Thoracoscopy.

## Data Availability

The data and supportive information are available within the article.

## LIST OF ABBREVIATIONS

CD: = Castleman Disease
PC: = Plasma Cell
MCD: = Multicentric CD

## STANDARDS OF REPORTING

PRISMA guidelines and methodology were followed.

## SUPPLEMENTARY MATERIAL

PRISMA checklist is available as supplementary material on the publisher’s website along with the published article.
